# Oleanolic Acid and Ursolic Acid Improve Bone Properties and Calcium Balance and Modulate Vitamin D Metabolism in Aged Female Rats

**DOI:** 10.3389/fphar.2018.01435

**Published:** 2018-12-04

**Authors:** Sisi Cao, Xue-Lian Tian, Wen-Xuan Yu, Li-Ping Zhou, Xiao-Li Dong, Murray J. Favus, Man-Sau Wong

**Affiliations:** ^1^Department of Applied Biology and Chemical Technology, The Hong Kong Polytechnic University, Kowloon, Hong Kong; ^2^State Key Laboratory of Chinese Medicine and Molecular Pharmacology (Incubation), The Hong Kong Polytechnic University Shenzhen Research Institute, Shenzhen, China; ^3^Shenzhen Key Laboratory of Food Biological Safety Control, The Hong Kong Polytechnic University Shenzhen Research Institute, Shenzhen, China; ^4^Section of Endocrinology, Department of Medicine, The University of Chicago, Chicago, IL, United States

**Keywords:** oleanolic acid, ursolic acid, bone protection, calcium balance, vitamin D metabolism, aged rats

## Abstract

Oleanolic acid (OA) and ursolic acid (UA) are the major chemical constituents in *Fructus Ligustri Lucidi* (FLL), a kidney-tonifying Chinese herb that is previously shown to improve bone properties and enhance calcium balance in aged female rats. The present study was designed to study if OA and UA act as the active ingredients in FLL to exert the positive effects on bone and mineral metabolism in aged rats. Aged (13-month-old) Sprague-Dawley female rats were randomly assigned to four groups with oral administration of drug or vehicle treatment for 12 weeks: medium calcium diet (MCD, 0.6% calcium), high calcium diet (HCD, 1.2% calcium), MCD + FLL (700 mg/kg/day), MCD + OA (23.6 mg/kg/day) + UA (8.6 mg/kg/day). A group of mature (3-month-old) female rats fed with MCD was included as positive control. The results demonstrated that FLL and OA+UA increased bone mineral density and improved microarchitectural properties of aged female rats. The osteoprotective effects of FLL and OA+UA might be, at least in part, associated with their actions on enhancing calcium balance and suppressing age-induced secondary hyperparathyroidism in aged female rats. FLL and OA+UA also significantly induced renal CYP27B1 protein expression and OA+UA treatment decreased CYP24A1 mRNA and protein expressions in aged female rats. In addition, FLL and OA+UA significantly increased the promoter activity, mRNA and protein expressions of renal CYP27B1 *in vitro* in human proximal tubule HKC-8 cells. The present findings suggest that OA+UA can be regarded as the active ingredients of FLL and might be a potential drug candidate for prevention and treatment of osteoporosis.

## Introduction

Osteoporosis is a skeletal disorder characterized by low bone mass and microarchitectural deterioration of bone tissue which increases the risk of fracture. In China, the prevalence of osteoporosis is 50% in women and 22.5% in men aged > 50 years (Wang et al., 2009). It is estimated that 40% of Chinese women and 10% of Chinese men are expected to have a major osteoporotic fracture after turning 50 years old (Si et al., 2015). For management of osteoporosis, calcium, vitamin D supplements, and pharmacological agents, such as bisphosphonates, teriparatide, and denosumab, are commonly used in the elderly. Although they exert beneficial effects on bones, the concerns about their potential side effects and safety of long-term use are rising (Tu et al., 2018). Given the rapid growth of the aging population, development of alternative approaches for management of bone health is pressing.

During the aging process, the combined effects of impaired calcium homeostasis, age-related decline in renal 1,25(OH)_2_D_3_ production, and vitamin D deficiency-induced secondary hyperparathyroidism contribute to an increase in the rate of bone loss (Veldurthy et al., 2016). 1,25(OH)_2_D_3_, the active form of vitamin D, is essential for intestinal calcium absorption and normal bone mineralization. In the renal proximal tubule, 1,25(OH)_2_D_3_ is synthesized by its biosynthetic enzyme 25-hydroxyvitamin D 1-α-hydroxylase (CYP27B1) and degraded by its catalytic enzyme 25-hydroxyvitamin D 24-hydroxylase (CYP24A1) (Bikle, 2014). The regulation of vitamin D metabolism is a tightly controlled process, in which CYP27B1 and CYP24A1 are regulated by a number of hormonal factors, including PTH (Zierold et al., 2003; Bikle, 2014), 1,25(OH)_2_D_3_ itself (Wang et al., 2015), fibroblast growth factor 23 (FGF-23) (Shimada et al., 2004; Ranch et al., 2011), as well as minerals such as calcium and phosphate ions (Wong et al., 1997, 2000). The age-related defect in 1 α hydroxylation by CYP27B1 (Armbrecht et al., 2007) and accelerated catabolism of 1,25(OH)_2_D_3_ by the increased CYP24A1 (Johnson et al., 1995) contribute to the decline in renal 1,25(OH)_2_D_3_ production with aging.

Oleanolic acid (3β-hydroxyolean-12-en-28-oic acid) and UA (3β-hydroxyurs-12-en-28-oic acid) are a pair of isomers that are found to be the ubiquitous triterpenoids in medicinal herbs and in the plant kingdom. In the past decade, increasing evidence suggests that OA and UA exert bone-protective effects. OA glucosides and OA derivatives has been reported to reduce osteoclast formation (Li et al., 2005, 2009; Zhang et al., 2005). Similarly, a quinoxaline derivative of OA was shown to prevent ovariectomized (OVX)-induced bone loss in both OVX mice (Zhao et al., 2011) and OVX rat models (Wu et al., 2018). Our most recent study showed that OA improved bone properties partially associated with its regulatory action on calcium-vitamin D axis in both aged female rats and mature OVX mice (Cao et al., 2018). On the other hand, UA was also shown to stimulate osteoblast differentiation in mouse osteoblastic MC3T3-E1 cells (Lee et al., 2008) and primary culture of rat calvarial osteoblasts (Porwal et al., 2017). FLL, an OA- and UA-rich Chinese herbal medicine, was first reported by our group that exerts positive effects on regulating bone turnover markers and enhancing calcium balance in mature OVX rats (Zhang et al., 2006). In fact, OA and is being used as the chemical markers for quality evaluation of FLL preparations (China Pharmacopoeia Committee, 2010). Our subsequent studies showed that FLL improved bone properties and calcium balance in both aged OVX (Zhang et al., 2008a,c) and aged ovary-intact rats (Zhang et al., 2008b). The positive effect of FLL on bone appears to be associated with its actions on inducing circulating 1,25(OH)_2_D_3_ level (Dong et al., 2010) and modulating serum PTH level (Dong et al., 2012). These studies clearly demonstrate that FLL protects bone through modulating calcium-vitamin D axis in mature and aged rat models. Therefore, it is of interest to study whether the combination of OA and UA, the major bioactive ingredients in FLL, accounts for its positive actions on bone health.

The present study was designed to determine if the combination of OA and UA could mimic the effect of FLL on protecting against age-related bone loss and to explore the potential mechanisms involved in their actions on regulating vitamin D metabolism. To test this hypothesis, the effects of FLL and OA+UA on bone, calcium balance, and vitamin D metabolism in aged female rats, as well as their direct regulatory effects on vitamin D metabolic enzymes in human proximal tubule (HKC-8) cells were investigated.

## Materials and Methods

### Drugs and Chemicals

Oleanolic acid and UA (both purity > 98%) were purchased from Shanghai Winherb Medical Technology Co. (Shanghai, China). The dried and powdered (20 kg) crude plant of FLL was purchased from Anhui province of China. The ethanol extract of FLL was prepared as previously reported (Zhang et al., 2006, 2008a; Dong et al., 2010). In brief, the powdered FLL was extracted with 70% ethanol for twice with each extraction period of 2 h. The preparation was filtered, and vacuum concentrated until almost all the ethanol was evaporated. A viscous residue was generated at the yield of 34.0%, calculated by weight of the starting dried powder of crude FLL. The contents of OA (33.7 mg OA/g of FLL) and UA (12.3 mg UA/g of FLL) in FLL was measured to authenticate the quality of FLL extract based on methods described previously (Zhang et al., 2006). According to Chinese Pharmacopeia (China Pharmacopoeia Committee, 2010), such quantities of OA and UA present in the extract suggest that the quality of the FLL extract is high. The viscous residue was freeze-dried for 3 days, vacuum packed, and stored in desiccators until further usage.

### Animal Study Design

Thirty-two retired breeder Sprague-Dawley (SD) rats aged 9 months (290–350 g) and 10 virgin female SD rats aged 3 months (220–250 g) were purchased from Beijing Vital River Laboratories (Beijing, China). The retired breeders were raised to 13-month old to serve as aged rat model before the purchase of the mature rats (3-month-old, *n* = 10) which serve as young positive control group. All rats were housed in a light-controlled (12 h light and dark cycle) and temperature-controlled (22°C) environment. Before treatment regimen, rats were pair fed with MCD (TD 98005, 0.6% calcium, 0.65% phosphorus) for 5 days as acclimation. Aged female rats were randomly assigned to four groups (*n* = 8/group) with orally administrated drug treatment for 12 weeks: MCD, HCD (TD 05005, 1.2% calcium, 0.65% phosphorus), MCD + FLL (700 mg/kg/day), and MCD + OA (23.6 mg/kg/day) + UA (8.6 mg/kg/day). HCD is included as a positive control group. Diets were purchased from Harlan Teklad (Envigo, Madison, WI, United States). The dosage of FLL extract and calcium content of the diets were the same as those used in our previous studies (Zhang et al., 2006, 2008a; Dong et al., 2010). The dosage of OA and UA were determined by their actual amount presented in FLL ethanol extract. As the natural amount of UA presented in FLL extract is only one-third of that of OA, we speculated that the low abundancy of UA might not contribute to the bone protective effect by itself. Instead of an OA or UA treatment group, we designed a mixture of OA and UA group to determine whether OA+UA mimics the actions of FLL. All animals were pair fed with 15 g/day, the minimal mean food intake of all groups, of MCD or HCD (for HCD group only) with water supply *ad libitum*. The leftovers were collected and recorded. The body weight of the rats was monitored weekly throughout the study period. Before sacrifice, rats were individually placed into metabolic cages for 24-h urine and feces collection. Upon sacrifice, blood was taken from abdominal aorta and serum was separated by centrifugation. Duodenal mucosa was harvested by scraping and placed in 1 mL Trizol^®^ reagent (Invitrogen, Carlsbad, CA, United States). Cortex of the left kidney was separated from medulla and frozen in liquid nitrogen. Serum in aliquots, duodenal mucosa, and kidney cortex were stored at -80°C for further analysis. The left tibia, femur, and the intact lumbar vertebra were collected with soft tissues cleaned, wrapped by phosphate buffered saline (PBS)-soaked gauze, and stored at -20°C for micro-CT scanning first and bone calcium content measurement afterward. Husbandry of animals was based on Guide for the Care and Use of Laboratory Animals (National Research Council [US], 2011) and the experimental procedure was approved by the Animal Ethics Committee of The Hong Kong Polytechnic University (ASESC No.: 130602).

### Biochemical Assays of Serum and Urine Samples

The concentration of calcium and phosphorous in both serum and urine samples were measured by standard colorimetric methods using commercial kits according to the manufacturer’s instruction (STANBIO Laboratory, Boerne, TX, United States). Urinary calcium and phosphorous were corrected by urinary creatinine (Cr) levels, which was determined by picric acid methods using commercial kits (Zhongsheng Beikong Bio-technology and Science Inc., Beijing, China). Serum PTH level was determined by Rat BioActive Intact PTH ELISA Kit (Immutopics, Inc., San Clemente, CA, United States) following the manufacturer’s protocol. Serum 1,25(OH)_2_D_3_ was extracted and measured according to the manufacturer’s protocol (Immundiagnostik AG, Bensheim, Germany). In brief, 1,25(OH)_2_D_3_ was extracted by diisopropyl ether for four times from the chromabond column. The eluate was then loaded on to the silica cartridges and washed by 4:96 isopropanol:hexane for five times and 6:94 isopropanol:hexane for three times. 1,25(OH)_2_D_3_ was eluted from the silica cartridge by 25:75 isopropanol:hexane and measured by 1,25(OH)_2_Vitamin D ELISA Kit (Immundiagnostik AG, Bensheim, Germany).

### Calcium Balance Study and Bone Calcium Content

The left tibia (after micro-CT scanning and with soft tissue removed) and 24 h fecal sample were first dried at 110°C in muffle furnace for 12 h and then ashed at 800°C for 20 h. The ash weight was recorded. To determine the calcium contents, 100 mg of bone/fecal ash was weighed and dissolved overnight in 2 mL of 37% HCl. The samples were diluted with Milli-Q water and analyzed by atomic absorption spectrophotometer (PerkinElmer, AAnalyst 100 Spectrometer, Norwalk, CT, United States). Calcium absorption and balance were calculated by the following equations: Ca absorption rate (%) = (Ca intake - fecal Ca)/Ca intake × 100; Ca balance = Ca intake - (urinary Ca + fecal Ca).

### Microcomputed Tomography (μCT)

Left tibia, left femur, and lumbar vertebra (L2) from each rat were scanned with micro-computed tomography (μCT) scanner (VivaCT40, Scanco Medical, Bassersdorf, Switzerland). Scans were performed at medium resolution and using energy of 70 kVp, intensity of 114 μA, with an integration time of 300 ms. Distal femur and proximal tibia were scanned in 210 slices from metaphyseal growth plate, in which the volume of interest was contoured for 100 continuous slices starting from the disappearance of the condyle (corresponding to a 2.1 mm region) for evaluation. For lumbar vertebra (L2), 150 slices centered in L2 (middle point ± 75 slices) were evaluated. Morphometric parameters include BMD (mg HA/cm^3^), BV/TV (%), Tb.N (1/mm), Tb.Th (mm), Tb.Sp (mm), and Conn.D (1/mm^3^) were calculated by using a three-dimensional direct model with a constant threshold of 300.

### Cell Culture

Human proximal kidney tubule cells (HKC-8) were a kind gift from Dr. Racusen of Johns Hopkins University (Racusen et al., 1997). HKC-8 cells were maintained in DMEM/F12 medium (Life Technologies, Carlsbad, CA, United States) supplemented with 5% fetal bovine serum (FBS) and 1X penicillin/streptomycin (Life Technologies). The cells were cultured on 100 mm culture dish in the 37°C incubator with 5% CO_2_-95% air atmosphere. 24 h before drug treatment, culture medium was changed to a chemical defined serum-free medium containing the following supplements (Life Technologies): insulin (5 μg/mL), transferrin (5 μg/L), Na_2_SeO_3_ (5 ng/mL), tri-iodothyronine (0.37 nmol/L), epidermal growth factor (2.5 ng/mL), and hydrocortisone (1 nmol/L). For assessment of promoter activities, mRNA, and protein expressions, HKC-8 cells were treated with vehicle (0.1% ethanol), 10^-5^ M forskolin or 10^-8^ M 1,25(OH)_2_D_3_, 100 μg/mL FLL ethanol extract, 10^-9^ M, 10^-7^ M, 10^-5^ M OA+UA (3:1) for 24 h. Forskolin (FSK, purity ≥ 98%, Sigma-Aldrich, St. Louis, MO, United States) and 1,25(OH)_2_D_3_ (1,25D, purity ≥ 99%, Sigma-Aldrich) were used as the positive controls for measuring CYP27B1 and CYP24A1 expressions, respectively.

### Real-Time Polymerase Chain Reaction (PCR) Analysis

Total RNA isolation of kidney cortex and cell samples was performed by using TRIzol^®^ Reagent (Life Technologies) following the manufacturer’s instructions. RT-PCR was performed by using M-MLV Reverse Transcriptase (Life Technologies) with oligo(dT)_12-18_ primers (Thermo Scientific, Waltham, MA, United States). Real-time PCR was carried out in 20 μL reaction mixture containing 10 μL Power SYBR^®^ Green PCR Master mix (Applied Biosystems), 0.5 μL cDNA template, and 250 nM of each primer using the ABI 7900HT Fast Real-Time PCR system (Applied Biosystems). Glyceraldehyde-3-phosphate dehydrogenase (GAPDH) was used as endogenous control. The primer sequences were the same as previously described (Cao et al., 2018). The relative quantity of mRNA was calculated using the SDS software package (Applied Biosystems, Carlsbad, CA, United States) by fitting the Ct value to the standard curve and each gene expression was normalized by its own GAPDH expression level.

### Western Blot Analysis

The cortex of left kidney and HKC-8 cells were homogenized and lysed in Nonidet P-40 Lysis Buffer supplemented with protease inhibitors as described previously (Dong et al., 2010; Cao et al., 2018). Protein concentrations were measured by Bradford protein assay (Bio-Rad Laboratories, Philadelphia, PA, United States). Equal amount of cytosolic protein (50 μg for tissue and 15 μg for cells) were separated by sodium dodecyl sulfate polyacrylamide gel electrophoresis (SDS-PAGE) gel and transblotted to polyvinylidene difluoride (PVDF) membrane (Immobilon-P, Bedford, MA, United States). The blots were probed at 4°C for overnight with the primary antibodies: rabbit anti-CYP27B1 (1:500, Santa Cruz Biotechnology, Santa Cruz, CA, United States), rabbit anti-CYP24A1 (1:500, Santa Cruz), or mouse anti-β-actin (1:5000, Abcam, Cambridge, MA, United States). Then the blots were incubated for 2 h with corresponding IgG-HRP (horseradish peroxidase)-conjugated anti-rabbit (1:2000, Santa Cruz) or anti-mouse (1:3000, Cell Signaling Technology, Beverly, MA, United States) secondary antibodies. The HRP signal was developed by incubating the blots with enhanced chemiluminescence (ECL) substrate (Clarity^TM^ Western ECL Substrate, Bio-Rad, United States) for 5 min. The bounded antibodies were visualized by Lumi-Imager (Roche, Manheim, Germany) and quantified by using NIH ImageJ software. The signal intensity of the bands was presented as biochemical light units (BLU). β-actin was used as the internal control for equal loading.

### Transit Transfection

HKC-8 cells were co-transfected with reporter plasmids containing CYP27- or CYP24-promoter construct and the constitutive thymidine kinase promoter plasmid (pRL-TK, Promega, Madison, WI, United States). The CYP27-and CYP24-promoter constructs are reporter plasmids containing the full-length insert of 1,576 bp CYP27B1 promoter region and a 300-bp vitamin D responsive region of CYP24 promoter, respectively. The CYP27 plasmid was a kind gift from Dr. Farzana Perwad (at San Francisco) (Chanakul et al., 2013) and CYP24-promoter construct, generated by the late Dr. Jack Omdahl (Kerry et al., 1996), was kindly provided by Dr. JoEllen Welsh (University at Albany-SUNY) (Kemmis et al., 2006). Transfections were conducted in serum-free medium with 0.4 μg of CYP27- or CYP24-promoter plasmid together with 0.1 μg TK plasmid with FuGene HD transfection reagent (Promega). After 4 h transfection followed by 24 h drug treatment, cells were lysed and luciferase activity was determined by Dual Luciferase Reporter Assay System (Promega). The promoter activities of CYP27 or CYP24 were normalized to pRL-TK and were presented as relative luciferase units (RLU).

### Statistical Analysis

All data obtained were presented as Mean ± SEM (standard error of mean). The differences among groups of data were analyzed by GraphPad Prism Version 6.00 (GraphPad Software, La Jolla, CA, United States). The significance between different groups of means was evaluated by one-way analysis of variance (ANOVA). Post-test analysis of multiple comparisons was carried by Tukey’s test at a confidence level of 95%. *P*-value (probability value) under 0.05 was considered as statistically significant.

## Results

### Body Weight and Biochemical Parameters

As expected, 3-month-old mature rats significantly gained weight (33.4%) while the body weight of the aged groups remained unchanged during the experimental period (Table 1). Serum calcium and phosphorous levels remained unchanged in rats in response to all treatments. Mature rats have a much lower urinary calcium/creatinine (Ca/Cr) level compared to that in aged rats fed with the same diet (*p* < 0.001 vs. aged rat fed with MCD) and OA+UA treatment significantly (*p* < 0.01 vs. MCD) suppressed age-related urinary calcium loss in rats. The intervention groups did not alter the urinary phosphorous/creatinine (P/Cr) levels while HCD group significantly decreased urinary phosphorous excretion in aged rats (*p* < 0.001 vs. MCD). Serum 1,25(OH)_2_D_3_ was low in aged female rats when compared to that of mature rats (*p* < 0.001 vs. Mature). HCD further down-regulated serum 1,25(OH)_2_D_3_ level in aged rats (*p* < 0.001 vs. MCD) as expected. Neither FLL nor OA+UA treatment seemed to alter the serum 1,25(OH)_2_D_3_ level in aged rats fed MCD. On the other hand, serum PTH level was elevated in aged rats, possibly due to the age-related secondary hyperparathyroidism (*p* < 0.01 vs. Mature). FLL and OA+UA treatment significantly suppressed the age-induced serum PTH level in aged rats (*p* < 0.05 vs. MCD).

**Table 1:** Effects of FLL and OA+UA on body weight and biochemical parameters in aged female rats.

	Mature	MCD	HCD	FLL	OA+UA
**Body weight change** (%)					
Weight change, %	33.4 ± 5.1***	-1.2 ± 0.3	-0.7 ± 0.2	-1.8 ± 1.9	-2.3 ± 1.3
**Serum chemistry**					
s-Ca, mg/dL	12.3 ± 0.3	11.8 ± 0.4	11.6 ± 0.2	12.6 ± 0.9	11.7 ± 0.5
s-P, mg/dL	5.0 ± 0.7	4.9 ± 0.3	5.7 ± 0.2	4.5 ± 0.2	4.7 ± 0.3
s-1,25D, pg/mL	34.3 ± 2.7***	21.0 ± 2.3	3.3 ± 0.6***	15.0 ± 4.3	13.7 ± 2.2
s-PTH, pg/mL	40.1 ± 5.7**	242.6 ± 34.6	219.2 ± 38.3	80.0 ± 15.6*	87.2 ± 9.1*
**Urine chemistry**					
u-Ca/Cr, mg/mg	0.07 ± 0.02***	0.32 ± 0.06	0.35 ± 0.03	0.21 ± 0.02	0.11 ± 0.04**
u-P/Cr, mg/mg	3.17 ± 0.38	2.37 ± 0.23	0.10 ± 0.06***	2.63 ± 0.36	1.55 ± 0.30
**Bone calcium content**					
Bone ash weight, mg	322.4 ± 5.0	308.3 ± 7.6	354.4 ± 5.9**	358.3 ± 11.5*	359.9 ± 5.4**
Ca/Ash bone, μg/mg	392.4 ± 3.1	393.2 ± 1.8	404.2 ± 3.5*	402.2 ± 1.7*	400.7 ± 6.4*

*A group of 3-month-old mature female rats (Mature, *n* = 10) and four groups of 13-month-old mature female rats (*n* = 8/group) were fed with medium calcium diet (MCD, 0.6% calcium, 0.65% phosphorous) or high calcium diet (HCD, 1.2% calcium, 0.65% phosphorous) and orally administrated with drug or vehicle treatment for 12 weeks: FLL (700 mg/kg/day) and OA (23.6 mg/kg/day) + UA (8.6 mg/kg/day). 1,25D, 1,25(OH)_*2*_D_*3*_; s, serum; u, urine; Ca, calcium; P, phosphorus; Cr, creatinine. Urinary Ca/Cr, urinary Ca to creatinine ratio; urinary P/Cr, urinary P to creatinine ratio. Body weight (% of change) from baseline to 12 weeks. Data were presented by mean ± SEM and analyzed by one-way ANOVA followed by Tukey’s multiple comparison test. ^∗^*P* < 0.05, ^∗∗^*P* < 0.01, and ^∗∗∗^*P* < 0.001 vs. MCD*.

### Bone Calcium Content and Bone Microarchitecture

*Fructus Ligustri Lucidi* and OA+UA significantly increased bone ash weight as well as the bone calcium content in the tibia of aged rats to a comparable level with those of HCD group (*p* < 0.05 vs. MCD, Table 1). Moreover, FLL and OA+UA treatments significantly increased BMD and improved bone microarchitecture at proximal tibia, distal femur and lumbar vertebra L2 of aged female rats (Table 2). Trabecular BMD of tibia, femur as well as spine were significantly reduced in aged rats when compared to those of mature rats (*p* < 0.001 vs. Mature). Such age-related bone loss in rats was prevented by treatments with HCD, FLL, and OA+UA at all three bone sites measured (vs. MCD). FLL and OA+UA significantly increased trabecular BMD at the proximal tibia in aged rats by 56% and 70%, at the distal femur by 28% and 32%, and at L4 by 45% and 39%, respectively (vs. MCD). In addition, the bone microarchitectural properties at the three sites were altered in aged rats, as revealed by the decreased BV/TV, Tb.N, Tb.Th, Conn.D, and increased Tb.Sp (vs. Mature). Treatment with HCD, FLL, and OA+UA significantly rescued the bone microarchitectural deterioration, as indicated by the induced BV/TV, Tb.N, Tb.Th, Conn.D, and suppressed Tb.Sp in aged rats (vs. MCD).

**Table 2:** Effect of FLL and OA+UA on bone mineral density (BMD) and bone microarchitecture at proximal tibia, distal femur, and lumbar vertebra L2 in aged female rats analyzed by micro-CT.

	Mature	MCD	HCD	FLL	OA+UA
**Proximal tibia**					
BMD, mg HA/cm^3^	488.9 ± 34.0***	208.5 ± 16.3	340.7 ± 15.2**	325.9 ± 29.1*	355.5 ± 23.9**
BV/TV, %	82.1 ± 3.1***	32.2 ± 2.8	51.7 ± 3.0*	49.3 ± 3.9*	53.8 ± 4.6*
Tb.N, mm^-1^	3.56 ± 0.15	3.11 ± 0.13	3.82 ± 0.08**	3.48 ± 0.05	3.65 ± 0.10*
Tb.Th, μm	290.4 ± 50.5**	105.0 ± 5.9	139.8 ± 7.3*	137.6 ± 11.1*	147.1 ± 11.4*
Tb.Sp, μm	55.0 ± 5.6***	215.9 ± 17.2	129.9 ± 10.4**	154.8 ± 12.6	128.0 ± 14.9*
Conn.D, mm^-3^	40.7 ± 5.1	32.2 ± 1.9	48.2 ± 1.8**	36.6 ± 1.0	40.3 ± 2.7*
**Distal femur**					
BMD, mg HA/cm^3^	475.5 ± 30.2***	278.2 ± 8.7	393.4 ± 17.4**	357.4 ± 24.7*	368.1 ± 25.0*
BV/TV, %	76.7 ± 3.1***	35.0 ± 3.7	60.0 ± 3.6**	52.0 ± 2.8*	52.9 ± 5.0*
Tb.N, mm^-1^	3.20 ± 0.15	2.93 ± 0.07	3.34 ± 0.03*	3.24 ± 0.01	3.25 ± 0.05
Tb.Th, μm	268.8 ± 36.2*	130.1 ± 4.2	191.2 ± 11.1*	156.6 ± 14.4	173.0 ± 19.2
Tb.Sp, μm	74.2 ± 6.2***	205.5 ± 0.5	124.6 ± 11.6***	152.7 ± 3.5*	146.3 ± 15.2**
Conn.D, mm^-3^	37.2 ± 3.3**	24.3 ± 1.8	31.2 ± 1.2*	31.4 ± 2.6*	33.8 ± 1.9**
**Lumbar vertebra**					
BMD, mg HA/cm^3^	452.4 ± 11.1***	277.4 ± 10.1	415.3 ± 18.2***	402.0 ± 33.7**	385.9 ± 28.4*
BV/TV, %	61.8 ± 2.0***	29.3 ± 1.3	51.0 ± 2.8**	48.1 ± 4.8*	47.6 ± 5.1*
Tb.N, mm^-1^	3.83 ± 0.05***	3.00 ± 0.08	3.56 ± 0.08***	3.42 ± 0.05**	3.62 ± 0.08***
Tb.Th, μm	162.1 ± 6.5***	100.1 ± 2.9	142.7 ± 10.2*	140.9 ± 14.9	139.2 ± 10.9*
Tb.Sp, μm	99.8 ± 4.8***	237.0 ± 9.8	137.4 ± 7.2***	151.7 ± 14.1***	140.2 ± 15.3***
Conn.D, mm^-3^	39.8 ± 1.6***	30.0 ± 1.2	39.7 ± 1.5**	35.3 ± 2.6	41.0 ± 2.3***

*A group of 3-month-old mature female rats (Mature, *n* = 10) and four groups of 13-month-old mature female rats (*n* = 8/group) were fed with medium calcium diet (MCD, 0.6% calcium, 0.65% phosphorous) or high calcium diet (1.2% calcium, 0.65% phosphorous) and orally administrated with drug or vehicle treatment for 12 weeks: FLL (700 mg/kg/day) and OA (23.6 mg/kg/day) + UA (8.6 mg/kg/day). Tibia, femur and lumbar vertebra (L2) were collected upon sacrifice and scanned by micro-CT system (viva-CT40, Scanco Medical, Switzerland). Bone microarchitecture parameters: bone mineral density (BMD), bone volume/tissue volume (BV/TV), trabecular number (Tb.N), trabecular thickness (Tb.Th), trabecular separation (Tb.Sp), structural model index (SMI), and connectivity density (Conn.D). Data were presented by mean ± SEM and analyzed by one-way ANOVA followed by Tukey’s multiple comparison test. ^∗^*P* < 0.05, ^∗∗^*P* < 0.01, and ^∗∗∗^*P* < 0.001 vs. MCD*.

### Calcium Balance Study

Mature rats had a lower fecal calcium excretion level, higher calcium absorption rate, and calcium balance than those of aged rats fed with MCD (*p* < 0.01 vs. MCD, Table 3), confirming the decrease in calcium absorption with aging. Such decrease could be ‘rescued’ by high calcium intake in aged rats. HCD significantly improved calcium balance in aged female rats (*p* < 0.05 vs. MCD). OA+UA significantly reduced fecal calcium loss (*p* < 0.05 vs. MCD) and increased calcium absorption rate (*p* < 0.05 vs. MCD) in aged female rats while the changes by FLL treatment did not reach statistical significance. Calcium balance was significantly improved in both FLL and OA+UA groups (*p* < 0.05 vs. MCD) after 12 weeks of treatment.

**Table 3:** Effects of FLL and OA+UA on calcium balance in aged female rats.

	Mature	MCD	HCD	FLL	OA+UA
Ca intake (mg/day)	89.1 ± 6.2	92.9 ± 4.2	181.9 ± 9.3	92.8 ± 5.6	93.1 ± 5.9
Urine Ca (mg/day)	0.6 ± 0.2	1.9 ± 0.7	5.7 ± 0.6***	2.8 ± 0.4	1.1 ± 0.4
Fecal Ca (mg/day)	43.3 ± 8.7**	78.4 ± 6.0	162.2 ± 17.6**	64.2 ± 6.7	63.3 ± 5.2*
Ca absorption rate (%)	48.1 ± 9.7**	11.5 ± 5.0	12.5 ± 9.7	28.7 ± 7.8	30.4 ± 5.1*
Net Ca balance (mg/day)	42.8 ± 8.8**	12.4 ± 2.4	27.8 ± 5.4*	29.1 ± 8.3*	28.8 ± 3.5*

*A group of 3-month-old mature female rats (Mature, *n* = 10) and four groups of 13-month-old mature female rats (*n* = 8/group) were fed with medium calcium diet (MCD, 0.6% calcium, 0.65% phosphorous) or high calcium diet (HCD, 1.2% calcium, 0.65% phosphorous) and orally administrated with drug or vehicle treatment for 12 weeks: FLL (700 mg/kg/day) and OA (23.6 mg/kg/day) + UA (8.6 mg/kg/day). 24 h urine and feces were collected before sacrifice. Ca absorption rate (%) = (Ca intake – fecal Ca)/Ca intake × 100; Ca balance = Ca intake – (urinary Ca + fecal Ca). Data were presented by mean ± SEM and analyzed by one-way ANOVA followed by Tukey’s multiple comparison test. ^∗^*P* < 0.05, ^∗∗^*P* < 0.01, and ^∗∗∗^*P* < 0.001 vs. MCD*.

### Renal CYP27B1 and CYP24A1 Gene and Protein Expressions in Aged Female Rats

**FIGURE 1:**
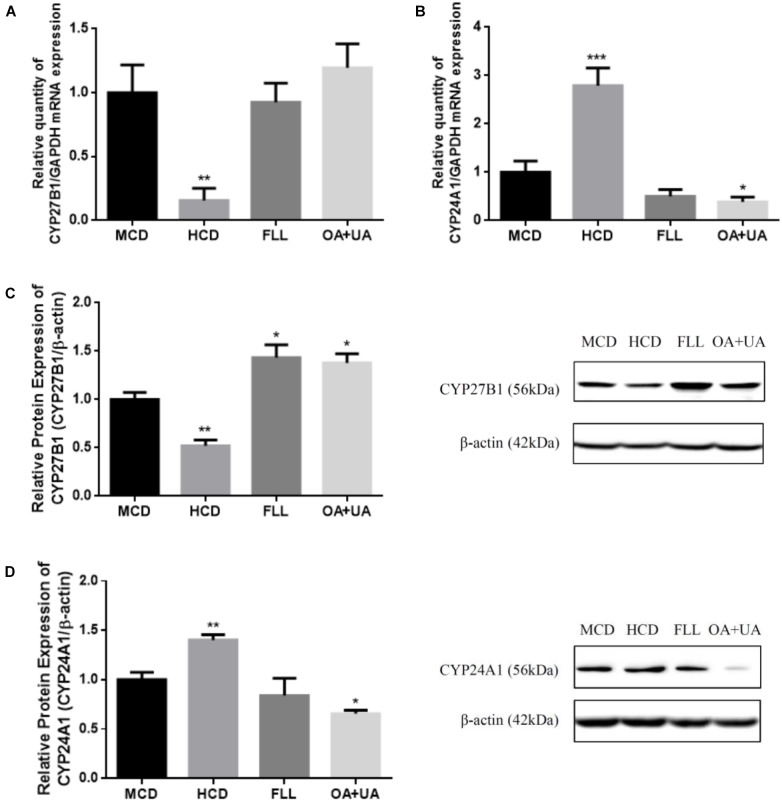
Effect of FLL and OA+UA on renal CYP27B1 **(A,C)** and CYP24A1 **(B,D)** mRNA and protein expressions in aged female rats. Thirteen-month-old mature female rats were fed with medium calcium diet (MCD, 0.6% calcium, 0.65% phosphorous) or high calcium diet (1.2% calcium, 0.65% phosphorous) and orally administrated with drug or vehicle treatment for 12 weeks: FLL (700 mg/kg/day) or OA (23.6 mg/kg/day) + UA (8.6 mg/kg/day). The renal mRNA expression of **(A)** CYP27B1 and **(B)** CYP24A1 were measured by real-time PCR analysis and the data were normalized with GAPDH. The renal protein expression of **(C)** CYP27B1 and **(D)** CYP24A1 were determined by western blot and the data were normalized with β-actin. Data were presented by mean ± SEM and analyzed by one-way ANOVA followed by Tukey’s multiple comparison test. ^∗^*P* < 0.05, ^∗∗^*P* < 0.01, and ^∗∗∗^*P* < 0.001 vs. MCD.

The effects of FLL and OA+UA on expression of renal vitamin D metabolic enzymes in aged female rats are shown in Figure 1. Aged rats fed with HCD have lower renal CYP27B1 mRNA (Figure 1A) and protein (Figure 1C) and higher CYP24A1 mRNA (Figure 1B) and protein (Figure 1D) expressions than mature rats as expected. FLL and OA+UA treatments significantly induced CYP27B1 protein expression (*p* < 0.05, Figure 1C), but not CYP27B1 mRNA expression (Figure 1A) in aged rats. In contrast, OA+UA treatment significantly suppressed the mRNA and protein expressions of renal CYP24A1 (*p* < 0.05, Figures 1B,D) in aged female rats, while the suppression by FLL treatment did not reach statistical significance.

### CYP27B1 and CYP24A1 Gene and Protein Expressions and Promoter Activities in HKC-8 Cells

**FIGURE 2:**
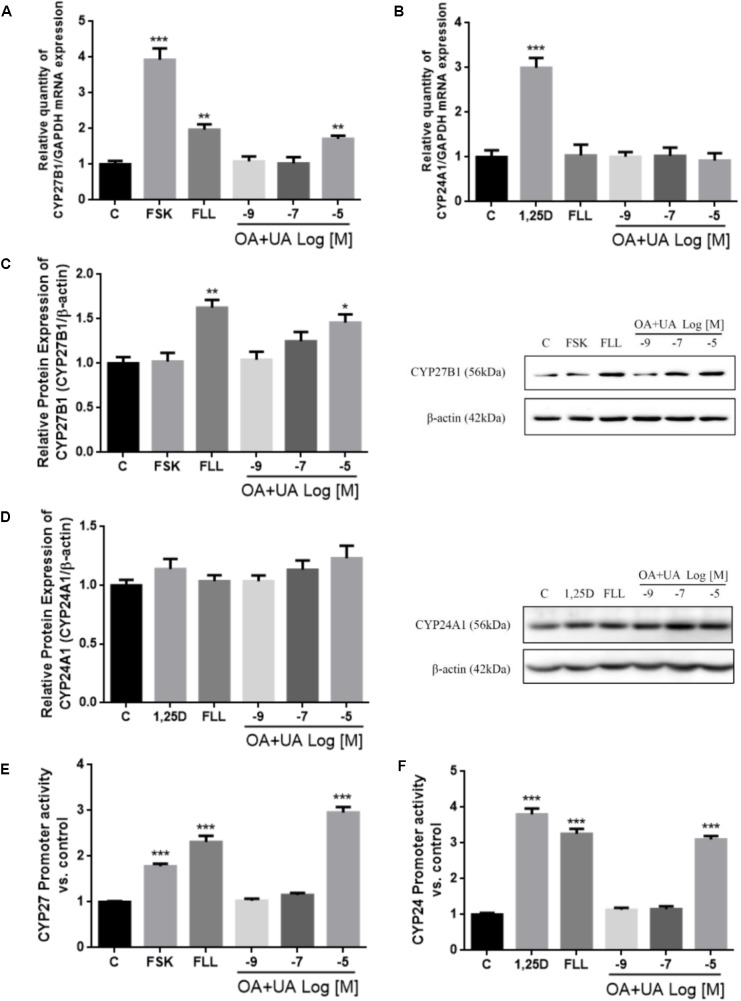
Effects of FLL and OA+UA on CYP27B1 and CYP24A1 mRNA, protein expressions, and promoter activities in HKC-8 cells. HKC-8 cells were treated with vehicle (0.1% ethanol), 10^-5^ M forskolin or 10^-8^ M 1,25(OH)_2_D_3_, 100 μg/mL FLL ethanol extract, 10^-9^ M, 10^-7^ M, 10^-5^ M OA+UA (3:1) for 24 h. The mRNA expression of **(A)** CYP27B1 and **(B)** CYP24A1 were measured by real-time PCR analysis and the data were normalized with GAPDH. The protein expression of **(C)** CYP27B1 and **(D)** CYP24A1 were determined by western blot and the data were normalized with β-actin. The promoter activity of **(E)** CYP27B1 and **(F)** CYP24A1 were measured by dual luciferase assay and data were normalized with thymidine kinase reporter construct. All experiments were repeated for at least three times. Data were presented as mean ± SEM and analyzed by one-way ANOVA followed by Tukey’s multiple comparison test. ^∗^*P* < 0.05, ^∗∗^*P* < 0.01, and ^∗∗∗^*P* < 0.001 vs. control.

The *in vitro* effects of FLL and OA+UA on CYP27B1 and CYP24A1 expressions were studied using HKC-8 cells (Figure 2). Treatment with FLL (100 μg/mL) and OA+UA (10^-5^ M) for 24 h significantly induced CYP27B1 mRNA expression by 97% and 71%, respectively (*p* < 0.01 vs. control, Figure 2A). FLL (100 μg/mL) and OA+UA (10^-5^ M) also up-regulated CYP27B1 protein expression by 52% (*p* < 0.01 vs. control) and 33% (*p* < 0.05 vs. control, Figure 2C), respectively. In contrast, both interventions did not alter CYP24A1 mRNA (Figure 2B) nor protein expressions (Figure 2D) in HKC-8 cells. Furthermore, FLL (100 μg/mL) and OA+UA (10^-5^ M) significantly increased CYP27 promoter activities in transfected HKC-8 cells by 2.3-fold and 2.9-fold (*p* < 0.001 vs. control, Figure 2E), respectively. Similarly, FLL and OA+UA induced CYP24 promoter activities in HKC-8 cells (*p* < 0.05 vs. control, Figure 2F).

## Discussion

The present study demonstrates that oral administration of FLL or OA+UA for 12 weeks significantly reduced age-related bone loss and improved bone microarchitectural properties in aged female rats. The positive actions of FLL and OA+UA on bone are, at least in part, associated with their effects on calcium balance and vitamin D metabolism. The mechanism by which FLL and OA+UA alter vitamin D metabolism might be through their actions on CYP27B1 and CYP24A1 expressions *in vivo*.

Aged rats treated with FLL or OA+UA for 12 weeks had significantly higher BMD at proximal tibia, distal femur, and lumbar vertebra L2 than aged rats from the control group. Bone microarchitecture, including the BV/TV, Tb.N, Tb.Th, and Conn.D were significantly increased, and Tb.Sp was significantly decreased in aged rats by treatment with FLL and OA+UA. The results were consistent with a previous study by us (Zhang et al., 2008c; Cao et al., 2018) and others (Bian et al., 2012; Feng et al., 2014; Lyu et al., 2014). Our previous study reported that oral administration of FLL at 700 mg/kg/day for 12 weeks increased BMD and bone mineral content (BMC) at tibia, femur, and lumbar vertebra of aged normal and OVX rats (Zhang et al., 2008c). Our most recent study also showed that OA significantly increased BMD and improved bone microarchitecture in mature OVX mice upon treatment for 6 weeks and in aged rats upon treatment for 12 weeks at these bone sites (Cao et al., 2018). Feng et al. (2014) and Lyu et al. (2014) demonstrated that feeding FLL-enriched diets for 4 months significantly increased femoral BMD and improved trabecular microarchitecture in growing male and female rats, respectively. Bian et al. (2012) showed that oral administration of OA at 20 mg/kg/day for 3 months significantly improved microarchitecture at lumbar vertebra in OVX rats. Similarly, quinoxaline derivative of OA was shown to be effective in increasing femoral BMD in OVX mice (Zhao et al., 2011) and rats (Wu et al., 2018), while an UA-rich herb (*Eriobotrya japonica*) was effective in increasing lumbar vertebral BMD in OVX mice (Tan et al., 2014). Future study by evaluating mechanical properties and cortical bone properties would provide us more information of the effect of FLL and OA+UA on improving bone strength and reducing fracture risk.

The possible mechanisms that mediate the bone protective effects of FLL and OA+UA might involve their actions on inhibiting osteoclast formation (Li et al., 2005, 2009) and stimulating osteoblast differentiation (Lee et al., 2008; Zhang et al., 2008c; Zhao et al., 2011). Recent mechanistic studies reported that FLL, OA, and UA inhibited osteoclastogenesis through receptor activator of NF-κB ligand (RANKL) signaling pathway in RAW264.7 cells (Xu et al., 2016). Li et al. (2015) showed that FLL stimulated osteoblast proliferation and differentiation via MAPK and Akt signaling pathways in MC3T3-E1 cells while Shu et al. (2017) reported that OA promoted mesenchymal stromal cell osteogenic differentiation by inhibiting Notch signaling pathway. Thus, the results of the present study together with those reported previously suggest that OA+UA are bone protective agents that may account for the beneficial effects of FLL on bones.

FLL and OA+UA, apart from their positive effects on bone, significantly increased calcium balance in aged rats. This observation was in line with our previous studies that demonstrated the effects of FLL in enhancing calcium absorption and calcium balance in aged normal and OVX rats, and such action was independent of the estrogen status of female rats (Zhang et al., 2008a,b). Our recent study also showed that OA could improve calcium balance in aged female rats possibly by increasing calcium entry across brush border membrane (Cao et al., 2018). Our studies also reported that FLL and OA+UA could increase intestinal calcium absorption and the expressions of calcium transport proteins such as TPRV6 and CaBP9k in aged rats (Zhang et al., 2008b; Cao et al., 2018). Indeed, the positive effects of FLL and its water fractions on enhancing calcium balance in other animal models have been reported in studies by us and others, including mature OVX rats (Zhang et al., 2006), mature female rats (Dong et al., 2012), and growing rats (Feng et al., 2014). Our study further indicated that the increase in net calcium balance by OA+UA involved the suppression of urinary calcium loss and fecal calcium secretion in aged female rats. Moreover, the increase in net calcium balance by FLL and OA+UA was associated with their positive effect on bone calcium content. Thus, our results clearly indicate utilization of dietary calcium was increased and the release of calcium from bone was decreased in aged female rats upon treatment with FLL and OA+UA. As the effects of FLL and OA+UA on calcium balance in aged rats were comparable, our results suggest that OA+UA is indeed the bioactive constituents that account for the actions of FLL on calcium homeostasis.

It is well known that age-related secondary hyperparathyroidism plays an essential role in the pathogenesis of osteoporosis and is associated with accelerated bone loss and increased fracture risks (Lips, 2001). Our result showed that serum PTH levels were much higher in aged rats than in mature rats and were suppressed in aged rats in response to treatment with FLL by 67% and OA+UA by 64%. This finding is in agreement with our previous report that serum PTH levels were restored to the normal level by treatment with FLL in aged OVX rats fed with different levels of dietary calcium (Zhang et al., 2008a). Thus, our study suggests that the protection against age-related bone loss by FLL and OA+UA is in part mediated by their actions on suppressing secondary hyperparathyroidism. Indeed, PTH is a peptide hormone that plays an essential role in regulating calcium homeostasis, including its actions on intestinal calcium absorption [through modulating circulating 1,25(OH)_2_D_3_] and renal calcium re-absorption (Fleet and Schoch, 2010). Therefore, the improvement of calcium balance in aged female rats by FLL and OA+UA might also be mediated by their effects on modulating serum PTH levels. Recent studies by Zhang et al. (2015) reported that FLL and its water fraction modulated PTH level through antagonizing calcium-sensing receptor (CaSR) in kidney and parathyroid gland in OVX mice and diabetic mice models (Zhang et al., 2014; Sha et al., 2017). Thus, it is possible that FLL and OA+UA might suppress secondary hyperparathyroidism in aged rats via their actions on CaSR in kidney and parathyroid glands. Future study is needed to investigate the mechanism involved in mediating the effect of FLL and OA+UA on serum PTH.

Treatments of FLL and OA+UA did not alter circulating 1,25(OH)_2_D_3_ level upon intervention for 12 weeks. The result was unexpected as our previous study demonstrated that FLL induced the serum 1,25(OH)_2_D_3_ level in 11-month-old aged female rats (Zhang et al., 2008a). The discrepancy could be due to the remarkably suppressed PTH level by FLL and OA+UA treatments in aged rats in the present study. As PTH normally stimulate 1,25(OH)_2_D_3_ production (Gensure et al., 2005), it is possible that the effects of FLL and OA+UA on increasing 1,25(OH)_2_D_3_ level is counteracted by their actions in decreasing serum PTH level. In contrast, both intervention groups significantly induced renal CYP27B1 protein expression and OA+UA could also suppress renal CYP24A1 mRNA and protein expressions in aged rats. This observation is in line with our previous findings that FLL significantly induced CYP27B1 mRNA and protein expressions (Dong et al., 2010) and OA suppressed CYP24A1 mRNA and protein expressions (Cao et al., 2018) in aged rats. Thus, although FLL and OA+UA failed to alter circulating level of 1,25(OH)_2_D_3_ in aged rats, our results indicated that FLL and OA+UA could regulate vitamin D metabolism in aged rats via altering the expression of CYP27B1 and CYP24A1 in kidney. Furthermore, recent studies reported extra-renal CYP27B1 plays an essential role in paracrine and autocrine control of 1,25(OH)_2_D_3_ production (Anderson et al., 2005; Bikle, 2016). Thus, it is also possible that the positive effects of FLL and OA+UA on bone properties as well as calcium balance might also be mediated by their actions in modulating local production of 1,25(OH)_2_D_3_ in bone and duodenum in aged rats. Future study will be needed to explore the role of extra-renal CYP27B1 in mediating the beneficial effects of FLL and OA+UA *in vivo*.

Our *in vitro* study also showed that OA+UA in 10^-5^M mimicked the effects of 100 μg/mL of FLL in regulating the expressions of CYP27B1 and CYP24A1 in HKC-8 cells. In fact, based on the composition of this batch of FLL, 100 μg/mL of FLL is estimated to contain 0.73^∗^10^-5^M OA and 0.27^∗^10^-5^M UA and thus in agreement with the effective concentration of OA+UA used in the *in vitro*. Thus, the results further supported that OA+UA are the bioactive ingredients of FLL that responsible for its actions on modulating vitamin D metabolism. Specifically, our results showed that FLL and OA+UA could regulate CYP27B1 expression in HKC-8 cells at both transcriptional and translational levels, by increasing CYP27B1 mRNA and protein expression and stimulating CYP27B1 promoter activities. Their actions were similar to those reported for PTH and forskolin in HKC-8 cells (Brenza et al., 1998; Brenza and DeLuca, 2000). In contrast, neither FLL nor OA+UA altered CYP24A1 mRNA or protein expression in HKC-8 cells. Such results were different from those observed *in vivo* in which OA+UA significantly suppressed renal CYP24A1 mRNA and protein expressions in aged rats. The discrepancy might be due to the presence of other hormonal factors that affect vitamin D metabolism *in vivo* (Murayama et al., 1999) and that the properties of HKC-8 cell lines might not completely resemble the properties of renal proximal tubules cell *in vivo*. Both treatments also unexpectedly induced CYP24A1 promoter activity in HKC-8 cells in a way similar to those reported by others for the action of PTH in porcine proximal tubule AOK-B50 cells (Zierold et al., 2000). The discrepancy between the responses of transiently transfected CYP24A1 promoter activity and the endogenous CYP24A1 expression might be due to the difference in the composition of the promoter region of the transfected plasmid and those of the endogenous gene. The lack of 3′untranslated region or a more distal promoter sequence of the CYP24A1 promoter might account for the lack of inhibitory responses of transfected HKC-8 cells to treatment with FLL and OA+UA.

The mechanisms involved in the modulatory effect of FLL and OA+UA on vitamin D metabolism are yet to be determined. OA and UA have been reported to antagonize the effects of transforming growth factor β (TGF-β) (Yoshimura et al., 2003), thereby inhibiting epithelial cell growth (Roberts, 1998). However, it is unclear if TGF-b dependent signaling pathway is involved in mediating the actions of FLL or OA+UA on vitamin D metabolism. Furthermore, studies (Sultana, 2011; Zhang et al., 2014) also demonstrated the modulatory effects of FLL, OA, and UA on the serum level of FGF-23, a bone-derived peptide that acts as a potent regulator of phosphorus and 1,25(OH)_2_D_3_ metabolism (Shimada et al., 2004). FGF-23 targets at the FGF receptor-α-klotho complex to suppress the CYP27B1 and induce the CYP24A1 mRNA expression in kidney both *in vivo* and *in vitro* (Perwad et al., 2007). It might be possible that FLL and OA+UA regulate renal CYP27B1 and CYP24A1 expressions by suppressing FGF-23 levels or by antagonizing its receptor to in aged female rats. Future studies will be needed to investigate the underlying mechanisms that mediate the effects of FLL and OA+UA on vitamin D metabolism.

In summary, the present study demonstrated that treatment of FLL and OA+UA for 12 weeks protected against age-related bone loss, improved calcium balance, and suppressed secondary hyperparathyroidism in aged female rats. The results suggest that the mixture of OA and UA can be regarded as the bioactive ingredients of FLL that account for its effects on protecting bone, increasing calcium balance, and modulating vitamin D metabolism *in vivo*. This study provides evidence to support that OA+UA might be used as a novel orally administrated therapeutic agent for prevention and management of osteoporosis. Further study measuring bone mechanical properties would provide insight into the effects of treatment on bone strength. Future studies are needed to identify the molecular targets and delineate the mechanisms by which FLL and OA+UA exert beneficial effects on calcium balance and the circulating levels of calciotropic hormones.

## Author Contributions

SC designed the experiments, conducted the research, analyzed the data, and drafted the manuscript. X-LT assisted in animal feeding. W-XY assisted in cell culture study. L-PZ and X-LD assisted in sample processing. MF assisted in serum 1,25(OH)_2_D_3_ measurement. SC, X-LD, and M-SW designed the study and X-LD, MF, and M-SW commented on the draft. All authors read and approved the final version of the manuscript.

## Conflict of Interest Statement

The authors declare that the research was conducted in the absence of any commercial or financial relationships that could be construed as a potential conflict of interest.

## Abbreviations

1,25(OH)_2_D_3_: 1,25-dihydoxyvitamin D_3_
μCT: microcomputed tomography
ANOVA: analysis of variance
BMD: bone mineral density
BV/TV: bone volume/total volume
Ca: calcium
Conn.D: connectivity density
Cr: creatinine
CYP24A1: 25-hydroxyvitamin D-24 hydroxylase
CYP27B1: 25-hydroxyvitamin D-1α hydroxylase
FLL: *Fructus Ligustri Lucidi*
GAPDH: glyceraldehyde 3-phosphate dehydrogenase
HCD: high calcium diet
MCD: medium calcium diet
OA: oleanolic acid
P: phosphorous
PTH: parathyroid hormone
Tb.N: trabecular number
Tb.Sp: trabecular separation
Tb.Th: trabecular thickness
UA: ursolic acid
